# Assessing Neurokinematic and Neuromuscular Connectivity During Walking Using Mobile Brain-Body Imaging

**DOI:** 10.3389/fnins.2022.912075

**Published:** 2022-06-03

**Authors:** Mingqi Zhao, Gaia Bonassi, Jessica Samogin, Gaia Amaranta Taberna, Camillo Porcaro, Elisa Pelosin, Laura Avanzino, Dante Mantini

**Affiliations:** ^1^Movement Control and Neuroplasticity Research Group, KU Leuven, Leuven, Belgium; ^2^S.C. Medicina Fisica e Riabilitazione Ospedaliera, Azienda Sanitaria Locale Chiavarese, Genoa, Italy; ^3^Department of Neuroscience and Padova Neuroscience Center, University of Padua, Padua, Italy; ^4^Institute of Cognitive Sciences and Technologies—National Research Council, Rome, Italy; ^5^Centre for Human Brain Health and School of Psychology, University of Birmingham, Birmingham, United Kingdom; ^6^Department of Neuroscience, Rehabilitation, Ophthalmology, Genetics and Maternal Child Health, University of Genoa, Genoa, Italy; ^7^IRCCS Ospedale Policlinico San Martino, Genoa, Italy; ^8^Department of Experimental Medicine, Section of Human Physiology, University of Genoa, Genoa, Italy

**Keywords:** electroencephalography (EEG), electromyography (EMG), mobile brain-body imaging (MoBI), gait analysis, brain oscillations, motor control

## Abstract

Gait is a common but rather complex activity that supports mobility in daily life. It requires indeed sophisticated coordination of lower and upper limbs, controlled by the nervous system. The relationship between limb kinematics and muscular activity with neural activity, referred to as neurokinematic and neuromuscular connectivity (NKC/NMC) respectively, still needs to be elucidated. Recently developed analysis techniques for mobile high-density electroencephalography (hdEEG) recordings have enabled investigations of gait-related neural modulations at the brain level. To shed light on gait-related neurokinematic and neuromuscular connectivity patterns in the brain, we performed a mobile brain/body imaging (MoBI) study in young healthy participants. In each participant, we collected hdEEG signals and limb velocity/electromyography signals during treadmill walking. We reconstructed neural signals in the alpha (8–13 Hz), beta (13–30 Hz), and gamma (30–50 Hz) frequency bands, and assessed the co-modulations of their power envelopes with myogenic/velocity envelopes. Our results showed that the myogenic signals have larger discriminative power in evaluating gait-related brain-body connectivity with respect to kinematic signals. A detailed analysis of neuromuscular connectivity patterns in the brain revealed robust responses in the alpha and beta bands over the lower limb representation in the primary sensorimotor cortex. There responses were largely contralateral with respect to the body sensor used for the analysis. By using a voxel-wise analysis of variance on the NMC images, we revealed clear modulations across body sensors; the variability across frequency bands was relatively lower, and below significance. Overall, our study demonstrates that a MoBI platform based on hdEEG can be used for the investigation of gait-related brain-body connectivity. Future studies might involve more complex walking conditions to gain a better understanding of fundamental neural processes associated with gait control, or might be conducted in individuals with neuromotor disorders to identify neural markers of abnormal gait.

## Introduction

Gait is a common but rather complex activity that supports mobility in daily life. The successful performance of normal gait relies on rhythmic steps of left and right lower limbs in alternation, with arms and trunk providing stability and balance (Vaughan, 2003). This is mediated by the musculoskeletal system and requires sophisticated coordination of the nervous system (Alamdari and Krovi, 2017). The involvement of cortical and subcortical regions to support gait control has been documented by studies using neuroimaging techniques such as functional magnetic resonance imaging (fMRI), positron emission tomography (PET), and single photon emission computer tomography (SPECT) (Bakker et al., 2007a,b; Takakusaki, 2013, 2017; Hamacher et al., 2015). Electroencephalography (EEG), which measures changes in scalp potentials associated with neuronal electrical activity, can be used to directly examine neural dynamics during gait. Specifically, wireless EEG systems can be used in combination with kinematic and/or electromyography (EMG) sensors placed over the limbs for mobile brain/body imaging (MoBI) experiments (Jungnickel et al., 2019). EEG-based MoBI platforms record body signals and neural signals simultaneously, and can be useful for the study of gait-related brain dynamics.

The analysis of gait usually requires precise information on limb kinematics (Jasiewicz et al., 2006; Kotiadis et al., 2010). Normal gait consists of recurrent cycles that contain consecutive stance and swing phases for each lower limb (Vaughan, 2003; Schmeltzpfenning and Brauner, 2013; Alamdari and Krovi, 2017; Price et al., 2021). A stance phase starts with the corresponding foot striking on the ground (i.e., heel strike event) and ends with the toe of that foot detaching the ground (i.e., toe off event). The subsequent swing phase starts with the toe off event and ends with the heel strike event, which moves the body forward. Since the lower limbs move in alternation, the stance is further subdivided into a single support period where only one foot is in contact with the ground, and two double support periods, before and after the single support period, where two feet are supporting the weight of the body. These gait phases rely on coordinated contractions of lower limb muscles, which can be captured by surface EMG sensors (Cappellini et al., 2006; Agostini et al., 2010; Bonnefoy-Mazure and Armand, 2015). For instance, the vastus, the biceps femoris and the tibialis anterior of a heel-striking leg show stronger EMG activity during the double support phase to maintain the equilibrium while allowing forward progression. The vastus contracts during the initial part of the single support phase whereas the gastrocnemius is active during the whole single support phase. Also, the EMG signals of the tibialis anterior and the biceps femoris are strong throughout and at the end of the swing phase, respectively.

Gait-related EEG signals typically contain, in addition to neural signals, relatively large ocular/movement/myogenic artifacts. These artifacts can be effectively attenuated (Zhao et al., 2021) or corrupted data segments can be excluded (Gwin et al., 2011; Seeber et al., 2014, 2015; Nathan and Contreras-Vidal, 2016; Wagner et al., 2016; Oliveira et al., 2017) to ensure a reliable analysis of gait-related neural oscillations. These neural oscillations are typically distinguished in delta (1–4 Hz), theta (4–8 Hz), alpha (8–13 Hz), beta (13–30 Hz), and gamma (>30 Hz) oscillations. Several studies used EEG recordings to evaluate modulations of specific neural oscillations with respect to kinematic events and phases of the gait cycle. For example, modulations of neural oscillations across the gait cycle were found in EEG data collected using treadmill walking (Gwin et al., 2011). Further EEG studies on gait also differentiated the functional roles of neural modulations in beta, low gamma and high gamma bands (Seeber et al., 2014, 2015). Other studies reported distinct beta band oscillatory networks in subserving gait adaptation (Bulea et al., 2015), and suggested that the prefrontal, posterior parietal, and sensorimotor network activity underlies speed control during walking (Wagner et al., 2016). More recent studies observed correlates of power reduction in alpha and beta oscillations with increase of gait speed (Nordin et al., 2020), as well as gait-phase-independent neural activity during voluntary gait modifications (Yokoyama et al., 2021).

It should be considered that EEG-based MoBI platforms permit not only the study of brain activity during movement, but also of its relationships with body signals. Associations between brain and body signals are typically referred to as brain-body connectivity. Coherence analysis (Shaw, 1984) has been the first technique proposed to assess the link between movement and EEG dynamics, or neurokinematic connectivity (NKC) (Bourguignon et al., 2011, 2015; Piitulainen et al., 2013), and between EMG and EEG dynamics, or neuromuscular connectivity (NMC) (Bayraktaroglu et al., 2011; Gwin and Ferris, 2012). The first NKC and NMC experiments were conducted in controlled settings and mainly involved the contraction of limb muscles, but subsequent studies also extended such analysis to treadmill and free walking conditions (Petersen et al., 2012; Roeder et al., 2018). Mobile EEG measures are characterized by a larger contribution of movement-related artifacts, thereby reducing the sensitivity of coherence analyses for NKC and NMC assessment (Bourguignon et al., 2011, 2015; Bayraktaroglu et al., 2013). To address this problem, other brain-body connectivity measures were proposed, which rely on the analysis of temporal correlations between neural oscillations and peripheral signal envelopes (Bayraktaroglu et al., 2013; Dähne et al., 2014; Tanaka and Saga, 2019; Watanabe et al., 2020). It should also be noted that all the studies conducted so far quantified the relationships of body signals with EEG signals at the scalp level.

Notably, recent developments in the field of EEG data acquisition and analysis have permitted to perform a reliable source localization, i.e., the estimate of neural activity in the brain (Michel and Murray, 2012). Particularly, it has been shown that the application of high-density EEG (hdEEG) montages with more than hundred electrodes yields a fine spatial sampling of scalp potentials (Liu et al., 2015; Song et al., 2015; Seeck et al., 2017); furthermore, the implementation of advanced artifact attenuation approaches (Zhao et al., 2021) and head modeling solutions (Taberna et al., 2019a,b, 2021) permits a more accurate characterization of neural oscillations in the source space from hdEEG recordings. In a recent study, we have demonstrated that the application of these advanced solutions on hdEEG data collected in walking participants can support a finer characterization of neural oscillations in different phases of the gait cycle (Zhao et al., 2022). Yet, it remains to be evaluated if the increased spatial specificity brought by hdEEG in neuronal signal reconstruction can support the reliable assessment of gait-related NKC and NMC in the source space, and if this approach provides additional information as compared to previous studies that quantified NKC and NMC at the sensor level (Petersen et al., 2012; Roeder et al., 2018). To address these questions, we conducted an experiment in young healthy participants using a hdEEG-based MoBI platform and assessed gait-related brain-body connectivity in the source space. More specifically, we aimed to test whether brain-body connectivity is characterized by specific spatial patterns depending on the specific body sensors and the neural oscillations of interest, and to evaluate if the brain regions showing robust brain-body connectivity are those typically related to motor execution, or also to motor planning and coordination.

## Materials and Methods

In this study, we designed and used a MoBI platform to collect myogenic and kinematic body signals, as well as hdEEG recordings in a group of healthy participants during treadmill walking. With the resulting MoBI data, we evaluated the discriminative power of the myogenic and kinematic signals by analyzing temporal correlations of the signal envelopes across body sensors. We also assessed the frequency-wise NKC and NMC in selected regions of interest (ROIs) across body sensors by calculating correlations of the envelopes of body signals with the power of the neural signals in each frequency, and evaluated the connectivity levels separately for alpha (8–13 Hz), beta (13–30 Hz), and gamma (30–50 Hz) frequency bands. We finally extended the analysis to the whole brain and examined the connectivity images in the source space across body sensors.

### Experiment and Participants

The experiment included 24 young, healthy participants (14 females and 10 males, age 22–31 years), who were not affected by any brain-related injury/disease or any other medical condition. The experimental procedures were approved by the Ethics Committee of the Liguria Region, Italy (reference: 238/2019) and were conducted in accordance with the 1964 Helsinki declaration and its later amendments. An informed consent was obtained from each participant. The participant was equipped with the MoBI platform described below, and was asked to walk with normal and constant speed during the experiment (Gwin et al., 2011; Wagner et al., 2016) on a Forcelink treadmill (Motek Medical B.V., Houten, Netherlands). Data collection started after familiarization with the treadmill walking. The task design consisted of three blocks of walk, each lasting 2 min, with 1-min rest in between.

### Mobile Brain-Body Imaging Platform

**FIGURE 1:**
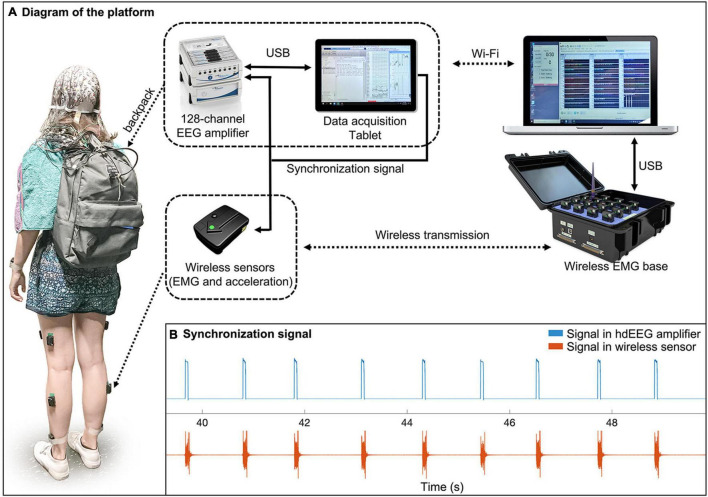
Diagram of the mobile brain-body imaging platform. **(A)** The platform consists of three parts: a backpack, a set of wireless body sensors, and a base station. The backpack contains a high-density EEG (hdEEG) amplifier, an EEG data acquisition tablet, and a light-weighted battery for powering the amplifier. During the experiment, a participant wears a hdEEG cap that is connected to the EEG amplifier in the backpack. The amplifier is connected to a tablet in the backpack for reliable EEG data recording. The EEG recording software on the tablet is remotely controlled by a laptop *via* Wi-Fi connection. The laptop is also connected to a wireless sensor base that receives EMG and acceleration data from sensors attached on the participant. The EEG system and wireless sensor system are synchronized by receiving a synchronization signal from the tablet through an auxiliary channel and one of the wireless sensors, respectively. **(B)** An example of synchronization signals recorded from the two systems.

The MoBI platform consisted of three main parts: *the backpack*; *the wireless body sensors*; and *the base station* (Figure 1A). *(1) The backpack* weighted about 1.7 kg and contained an ActiCHamp EEG amplifier (Brain Products GmbH, Gilching, Germany) with 128 channels, a Surface Go tablet (Microsoft Corporation, Redmond, WA, United States) and a light-weighted battery. The amplifier, powered by the battery, was connected to the tablet for reliable data storage. The EEG sensors, which were integrated in a cap with standard 10/20 montage, were connected to the subject’s scalp using a conductive gel. *(2) The wireless body sensors* were attached on the skin of the participant and permitted to collect simultaneous 3-axis acceleration and EMG signals. *(3) The base station* contained a 16-channel Trigno base (Delsys Inc., Natick, MA, United States) and a laptop. The Trigno base was used to receive acceleration/EMG data from the wireless sensors and to transfer them to the laptop through a USB cable connection. The laptop also controlled the Trigno base and the EEG data acquisition tablet for experimental flow management. A pulse signal (Figure 1B) was sent from the tablet to one of the auxiliary channels of the hdEEG amplifier and to one of the wireless sensors for an offline synchronization of the two systems. The temporal difference between the two was quantified using cross correlation (Supplementary Figure 1a). The temporal jitters after synchronization were below 5 ms (Supplementary Figure 1b).

### Data Collection

During the experiment, we collected 128-channel hdEEG signals at 1 kHz sampling rate, using the FCz electrode as physical reference. In addition, we collected 3-axis acceleration signals and EMG signals, respectively at 148 Hz and 2 kHz sampling rate, using wireless body sensors over the following bilateral muscles: vastus medialis, biceps femoris, tibialis anterior, gastrocnemius; using the same system, we also measured 3-axis acceleration signals from body sensors placed over the left and right ankles (Supplementary Figure 2). These anatomical locations were specifically chosen as they were reported to be related to walking movements (Winter and Yack, 1987; Liu et al., 2008; Bonnefoy-Mazure and Armand, 2015). The sensor positioning was performed in compliance with published guidelines (Hermens et al., 2000). Immediately after the experiment, we acquired a 3D scan of the participant’s head using an iPad (Apple Inc., Cupertino, CA, United States) equipped with a Structure Sensor camera (Occipital Inc., Boulder, CO, United States), to extract the locations of the hdEEG electrodes (Taberna et al., 2019a,b).

In a separate session, the structural MR image of each participant’s head was collected with a 3T Philips Achieva MR scanner (Philips Medical Systems, Best, Netherlands) using a T1-weighted magnetization-prepared rapid-acquisition gradient-echo (MP-RAGE) sequence. The scanning parameters were: repetition time (TR) = 9.6 ms, echo time (TE) = 4.6 ms, 160 coronal slices, 250×250 matrix, voxel size = 0.98 × 0.98 × 1.2 mm^3^.

### Analysis of Kinematic and Electromyographic Data

For each participant, we estimated the velocities using the integrals of left and right ankle total acceleration (Supplementary Figure 3). Total ankle acceleration signal segments with stable velocity were isolated and extracted to detect gait events and gait cycles in line with previous kinematic studies (Jasiewicz et al., 2006; Kotiadis et al., 2010). Accordingly, we detected the following gait events: left heel strike (LHS), right heel strike (RHS), left toe off (LTO), and right toe off (RTO) (Gwin et al., 2011; Seeber et al., 2014; Wagner et al., 2016). A full gait cycle was defined as the period between two adjacent left heel strikes (Zhao et al., 2022).

We then extracted trial-averaged velocity envelopes and EMG envelopes according to the acceleration signals and the EMG signals from the sensors on the limbs. Specifically, for each body sensor, the velocity was estimated from the integral of the total acceleration signal; the EMG signals were digitally filtered in the band [1–500] Hz and rectified (Türker, 1993; Rainoldi et al., 2004; Halliday and Farmer, 2010). The velocity envelopes and the EMG envelopes, as derived separately from the Hilbert transformation of the velocity and processed EMG signals, were resampled to 200 Hz, epoched based on the gait cycles, standardized to gait cycle percentage according to the corresponding RHS, LTO, and RTO events, and finally averaged for each participant.

The two types of trial-averaged envelopes were plotted for each body sensor as range curves (minimum to maximum range, intra-quartile range, and median curve) to examine the movements and the muscular activations of the limbs across the gait cycle. The inter-dependence of the envelopes was quantified using correlations across body sensors. The resulting correlation values were transformed to z-values using the Fisher transform, and then subject to one-sample *t*-tests to assess their statistical significance. The z-values were also averaged across participants; the resulting z-values were back-transformed to correlation values using the inverse Fisher transformation. These group-level correlation values were used for visualization purposes. Furthermore, the mean z-value and the maximum absolute z-value across body sensors were calculated for each subject, and the resulting values were visualized using box plots, separately for each signal type (velocity/EMG envelopes).

### Analysis of Neural Data

The hdEEG data analysis involved three main steps: EEG data preprocessing, head model creation, neural signal reconstruction. These steps will be described in the following sections.

#### Electroencephalography Data Preprocessing

We first corrected the bad channels in the hdEEG data (Guarnieri et al., 2018), digitally filtered them in the frequency band between 1 and 80 Hz and downsampled them to 200 Hz. Then, we applied a multi-step blind source separation approach to minimize the impact of ocular, movement and myogenic artifacts in the mobile hdEEG data (Zhao et al., 2021). Specifically, we attenuated the ocular artifacts by decomposing the hdEEG data with deflation-FastICA (Hyvarinen, 1999) and removing the components with maximum kurtosis over 5-s windows above 12; then, we attenuated the movement artifacts by decomposing the data with symmetric-FastICA (Hyvarinen, 1999) and removing the resulting components with mean sample entropy over 20-s windows below 0.8; finally, we attenuated the myogenic artifacts by decomposing the data with independent vector analysis (Anderson et al., 2011) and removing the components with power in the [30–80 Hz] band larger than the power in the [1–30 Hz] band. After artifact attenuation, we re-referenced the hdEEG signals using the average reference approach (Liu et al., 2015).

#### Head Model Creation

For each participant, a 12-layer realistic head volume conduction model was created for the neural activity reconstruction step. Specifically, three steps were followed: *electrodes position detection and coregistration*; *head tissue segmentation*; and *lead-field matrix calculation* (Taberna et al., 2021). *(1) Electrodes position detection and coregistration*. We detected the precise locations of the EEG electrodes using the SPOT3D toolbox, and coregistered them to the scalp of the individual MR image (Taberna et al., 2019a,b). *(2) Head tissue segmentation*. Using the MR-TIM software (Taberna et al., 2021), we segmented the individual MR image to 12 tissue layers: skin, eyes, muscle, fat, spongy bone, compact bone, cortical/subcortical gray matter, cerebellar gray matter, cortical/subcortical white matter, cerebellar white matter, cerebrospinal fluid, and brain stem. The conductivity value of each tissue layer was defined according to relevant studies (Haueisen et al., 1997; Holdefer et al., 2006). *(3) Lead-field matrix calculation*. The individual lead-field matrix projects the neural activity from the source space to scalp electric potentials. The matrix was calculated using the Simbio finite element method (Vorwerk et al., 2018), by meshing the tissue layers to 6-mm hexahedrons and placing source dipoles in the hexahedrons located inside the gray matter.

#### Neural Activity Reconstruction

We reconstructed the neural activity in the source space for each participant using the exact low-resolution brain electromagnetic tomography (eLORETA) algorithm (Pascual-Marqui et al., 2011), as in our previous hdEEG studies (Liu et al., 2017; Samogin et al., 2019; Zhao et al., 2019, 2021). This choice is corroborated by a comparative analysis performed on different source localization methods, which showed eLORETA to be particularly suitable for neural source signal reconstruction from hdEEG data (Liu et al., 2018). The preprocessed hdEEG data and the individual head model were fed to the eLORETA algorithm, resulting in an estimation of the oscillation strength and orientation of the dipole in each voxel of the gray matter, at each temporal moment. The estimated three-dimensional current density signal of each voxel was projected to a representative signal by taking the first principal components obtained from a principal component analysis (PCA).

### Brain-Body Connectivity Analyses

The reconstructed neural signals were analyzed in combination with kinematic and EMG data to assess frequency-dependent NKC and NMC, respectively. We initially assessed the NKC and NMC in a region of interest (ROI) located in the right primary motor cortex (M1) (Supplementary Table 1). For each participant, we first transformed the MNI coordinates into individual coordinates, and extracted the signals from the voxels within a 6-mm sphere centered on the coordinates in individual space. Then, a representative ROI signal was calculated as the first principal component of the signals in the different ROI voxels. We calculated a spectrogram in the frequency range [1–50 Hz] by using a continuous wavelet transformation (number of octaves = 6, voices per octave = 8). The frequency-specific power modulations of the spectrogram were epoched, standardized and averaged according to the same procedure used for the velocity/EMG envelopes. This approach permitted the estimate of frequency-dependent neural power dynamics during gait. After, the correlation between neural power dynamics at each frequency and velocity/EMG envelopes in the gait cycle was calculated to quantify frequency-dependent NKC/NMC. Such analysis was conducted for the M1 region in both hemispheres. Specifically, brain-body connectivity was assessed for ROIs that were either ipsilateral or contralateral to the body sensor, in alpha (8–13 Hz), beta (13–30 Hz), and gamma (30–50 Hz) bands, respectively. The connectivity values were also analyzed statistically. Specifically, we tested for differences across body sensors, ROIs, frequency bands and body signal types, using a four-way analysis of variance (ANOVA). To test whether gait-related brain-body connectivity was not only present in brain regions associated in motor execution, but also motor planning and coordination, we extended the ROI analysis to bilateral regions in thalamus (THAL), premotor cortex (PMC), posterior parietal cortex (PPC), and cerebellum (CER) (Supplementary Table 1). For each ROI, we used a two-way ANOVA on the connectivity values, with body sensor and frequency band as factors; we also conducted additional statistical analyses using one-sample *t*-tests. Probabilities were corrected for multiple comparisons using the false discovery rate (FDR) method.

We finally conducted a brain-body connectivity analysis across brain voxels, by creating volumetric images for each participant. The individual connectivity images were warped to MNI space by applying a non-rigid deformation calculated from the head MR image of each participant (Zhao et al., 2019). The group-level connectivity patterns were obtained by averaging the images across participants for each frequency band, body sensor and signal type. To further examine the main effects and interaction of frequency band and body sensor, we applied a voxel-wise two-way ANOVA on the same connectivity images. The resulting statistical parametric images were thresholded at *p* < 0.001 with multiple comparison correction using FDR.

## Results

### Gait-Related Kinematic and Electromyographic Profiles

**FIGURE 2:**
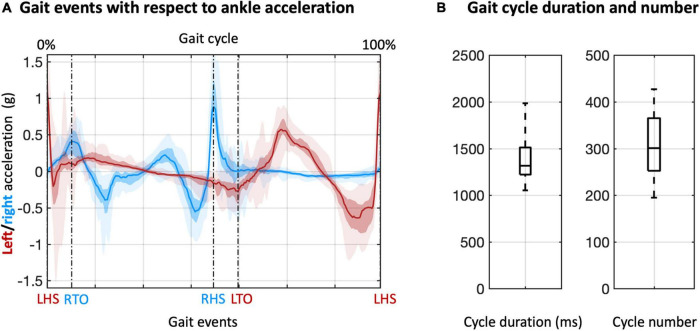
Detection and analysis of gait cycles. **(A)** Gait cycles were defined based on two adjacent left heel strike (LHS) events, detected from the acceleration of the left and right ankles. The acceleration data were also used to define right toe off (RTO), right heel strike (RHS), and left toe off (LTO) events. The lines denote the median across participants; the colored areas denote the intra-quartile range; the areas with semi-transparent color denote the full-range (from minimum to maximum) across participants. **(B)** Duration and number of gait cycles across participants.

**FIGURE 3:**
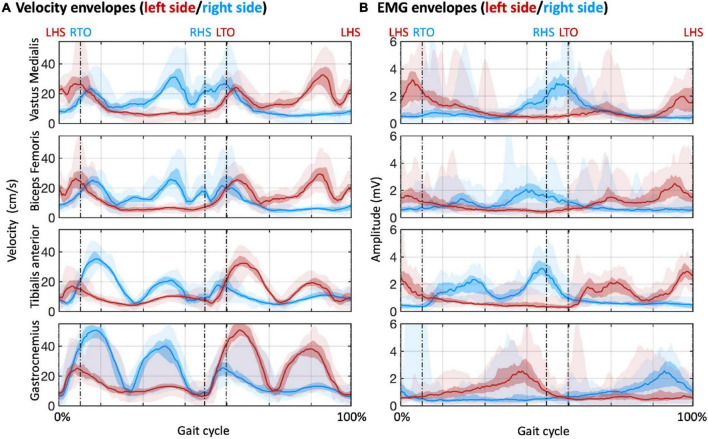
Velocity and EMG envelopes for sensors placed over selected muscles of the lower limbs. The muscles are the vastus medialis, biceps femoris, tibialis anterior, and gastrocnemius. **(A)** Velocity envelopes in the gait cycles across participants. **(B)** EMG envelopes in the gait cycles across participants. The lines denote the median across participants; the colored areas denote the intra-quartile range; the areas with semi-transparent color denote the full range (from minimum to maximum) across participants. Red and blue colors are used to indicate results for the muscles in the left and right sides of the body, respectively. LHS, left heel strike; RHS, right heel strike; LTO, left toe off; RTO, right toe off.

Our analysis started from the detection of the gait events and gait cycles (Figure 2). Clear gait-related modulations of left and right ankle acceleration signals were observed, with a consistent pattern across participants (Figure 2A). Based on the LHS events of the continuous steps, we detected about 200 to more than 400 gait cycles for each participant, with average cycle duration ranging from 1000 to 2000 ms (Figure 2B). With these gait events, we examined myogenic and kinematic signals of the body sensors across the gait cycle. Clear and consistent modulations both in velocity envelopes (Figure 3A) and EMG envelopes (Figure 3B) were observed across participants. The analysis of EMG envelopes evidenced contractions of ipsilateral leg muscles in different phases of the gait cycle. The vastus medialis contracted during a short period before and after the heel strike event. The biceps femoris and tibialis anterior activated mainly during the swing phase and around the heel strike event. The gastrocnemius contracted mainly in the stance phase. On the contrary, the velocity envelopes did not show substantial differences across ipsilateral sensors: the velocity fluctuated during swinging and kept flat during stance.

**FIGURE 4:**
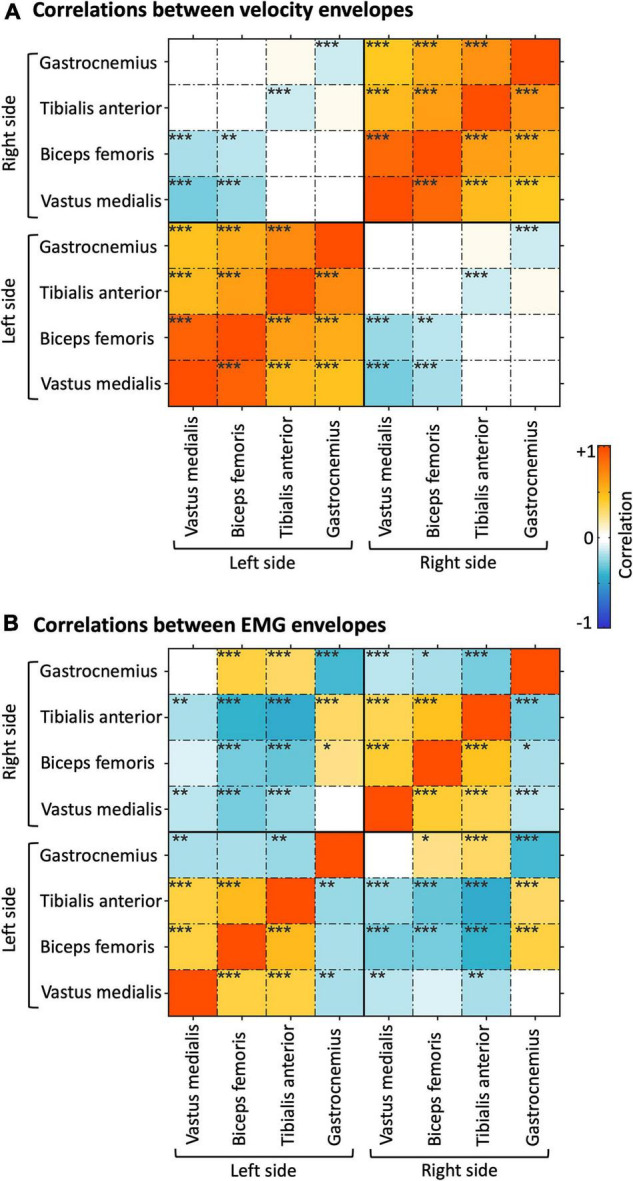
Correlation analysis of velocity and EMG envelopes. **(A)** Correlations of velocity envelopes across body sensors. **(B)** Correlations of EMG envelopes across body sensors. The results presented refer to the average across participants. *p_FDR_ < 0.05; **p_FDR_ < 0.01; ***p_FDR_ < 0.001.

**FIGURE 5:**
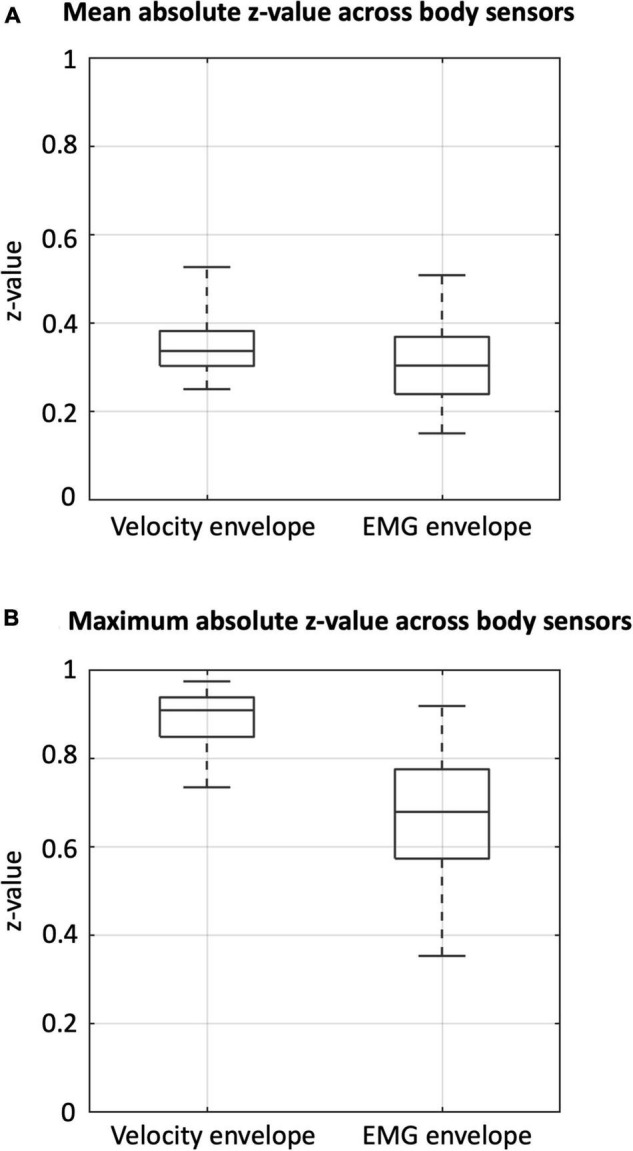
Inter-dependence of velocity and EMG envelopes across body sensors. Box plots indicating **(A)** the mean absolute z-value and **(B)** the maximum absolute z-value, for velocity and EMG envelopes, respectively.

To examine the cross-sensor inter-dependence of the velocity and the EMG envelopes, we calculated correlations across sensors, separately for the two signal types (Figure 4) and between them (Supplementary Figure 4). The velocity envelopes showed significant positive correlations in all the ipsilateral sensors, and a significant negative correlation between contralateral sensors for the thigh (i.e., vastus medialis and biceps femoris) (Figure 4A). The EMG envelopes of vastus medialis, biceps femoris, and tibialis anterior had significant positive correlation for the ipsilateral lower limb, and significant negative correlation for the contralateral lower limb (Figure 4B). Conversely, a reversed correlation pattern was found in the EMG envelopes of gastrocnemius (Figure 4B). We further analyzed the average level of inter-dependence of each of the two signal types (Figure 5A). This analysis revealed a higher inter-dependence between velocity envelopes (${\bar{r}}_{a\,b\,s\,\_\,m\,e\,a\,n}=0.35$) than EMG envelopes (${\bar{r}}_{a\,b\,s\,\_\,m\,e\,a\,n}=0.31$). The difference between signal types was more prominent when the maximum level of inter-dependence was considered (${\bar{r}}_{a\,b\,s\,\_\,m\,a\,x}=0.89$ and 0.68, respectively) (Figure 5B).

### Brain-Body Connectivity in Regions Related to Motor Planning, Execution, and Coordination

**FIGURE 6:**
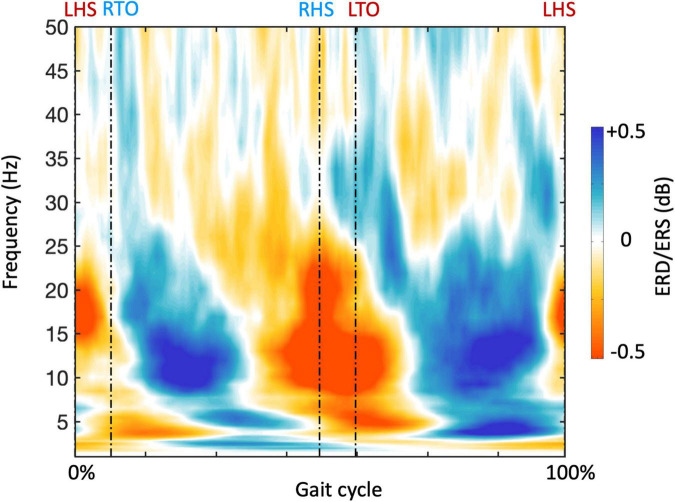
Gait-related time-frequency analysis for a representative region of interest. The plot shows the average frequency-dependent modulations of neural signals in the gait cycle across participants, calculated from the neural signal of the right primary motor cortex (M1). The timing of gait events is indicated using dashed vertical lines. LHS, left heel strike; RHS, right heel strike; LTO, left toe off; RTO, right toe off.

**FIGURE 7:**
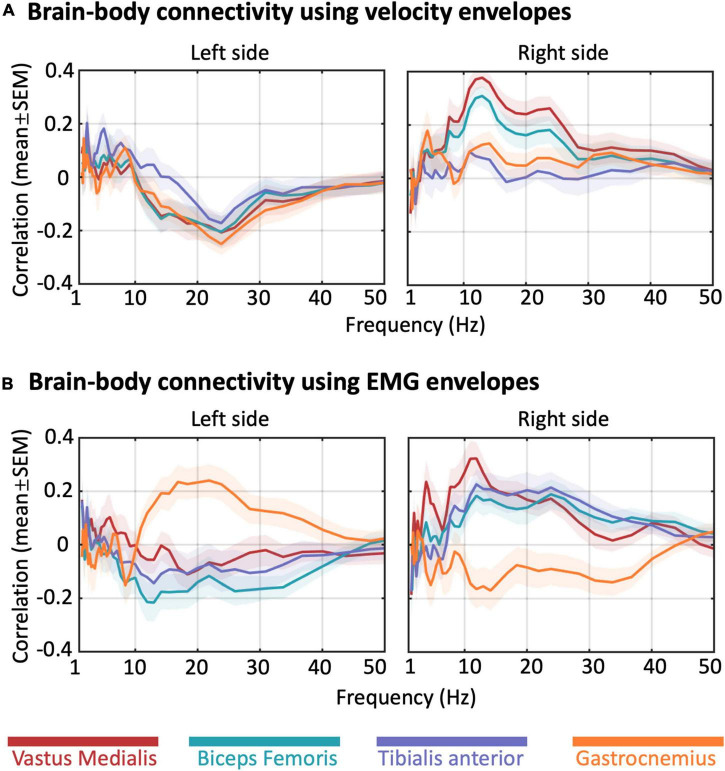
Frequency-dependent brain-body connectivity for a representative region of interest. The plots show the brain-body connectivity calculated from the neural signal of the right primary motor cortex (M1), using **(A)** velocity envelopes and **(B)** EMG envelopes, respectively. Colored lines denote the mean across participants, and colored areas denote the standard error of the mean.

In order to examine NKC and NMC, we reconstructed band-limited power envelopes of the neural signal for the right M1 region (Figure 6). The power in alpha and beta bands decreased during leg swings and rebounded after the swings, whereas a reverse modulation emerged for the gamma band. By calculating the correlation of the power signals in each frequency with each type of body signals, we generally revealed visible and stable NKC and NMC in the alpha and beta bands for the right M1 (Figure 7). The NKC were negative for all the left limb sensors (Figure 7A, left panel), and were positive for all the right limb sensors (Figure 7A, right panel). The NMC was negative for the vastus medialis, biceps femoris, and tibialis anterior located in the left lower limb, whereas it was positive for the gastrocnemius on the same limb (Figure 7B, left panel). A completely reversed connectivity pattern was observed for the sensors of the right leg (Figure 7B, right panel).

**FIGURE 8:**
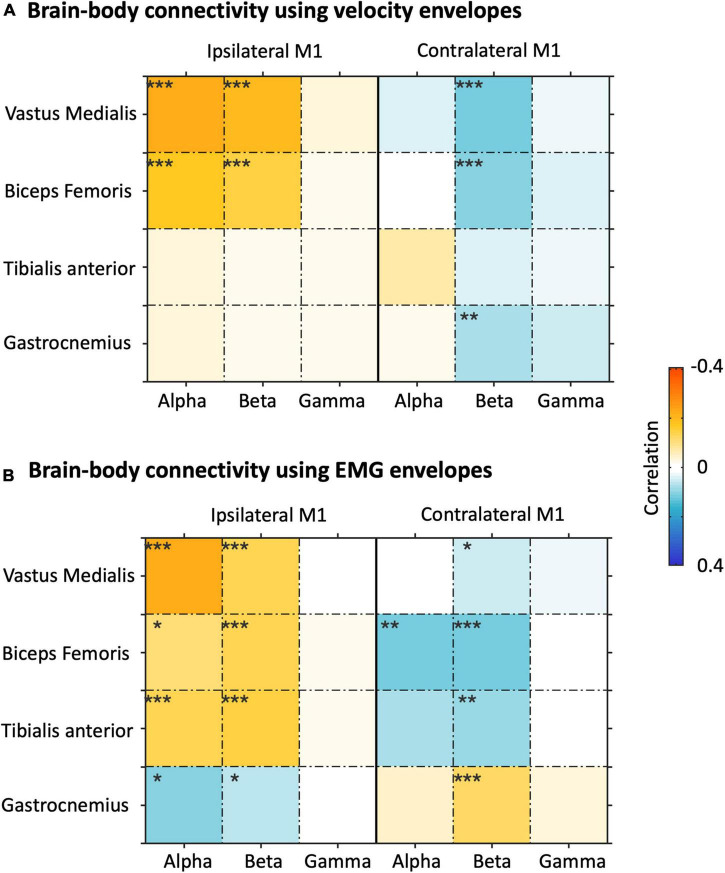
Analysis of brain-body connectivity for ipsilateral and contralateral primary motor cortex (M1), in alpha, beta, and gamma frequency bands. **(A)** Brain-body connectivity assessed using envelopes of reconstructed neural signals and velocity envelopes. **(B)** Brain-body connectivity assessed using envelopes of reconstructed neural signals and EMG envelopes. *p_*FDR*_ < 0.05;**p_FDR_ < 0.01; ***p_FDR_ < 0.001.

We then extended the NKC and NMC analysis also to the left M1 ROIs, and examined the connectivity matrices in alpha, beta, and gamma band for bilateral M1 ROIs (Figure 8). In general, strong and significant brain-body connectivity was present in the alpha and beta bands, whereas less reliable connectivity was found in the gamma band. The NKC of all the sensors was positive in the ipsilateral M1 and was negative in the contralateral M1, with relatively fewer sensors showing significant values as compared to the NMC (Figure 8A). For the vastus medialis, biceps femoris, and tibialis anterior, the NMC was positive in the ipsilateral M1 and was negative in the contralateral M1 (Figure 8B). Conversely for the gastrocnemius, the ipsilateral M1 showed negative connectivity and the contralateral M1 showed positive connectivity. To test the relative contribution of body sensor, ROI, frequency band, and body signal type on the connectivity values, we run a four-way ANOVA (Supplementary Table 2). The results revealed significant differences in connectivity across body sensors, ROIs and frequency bands (*p* < 0.001 for each of the three factors). Conversely, we found no significant differences between body signal types (*p* = 0.806).

**FIGURE 9:**
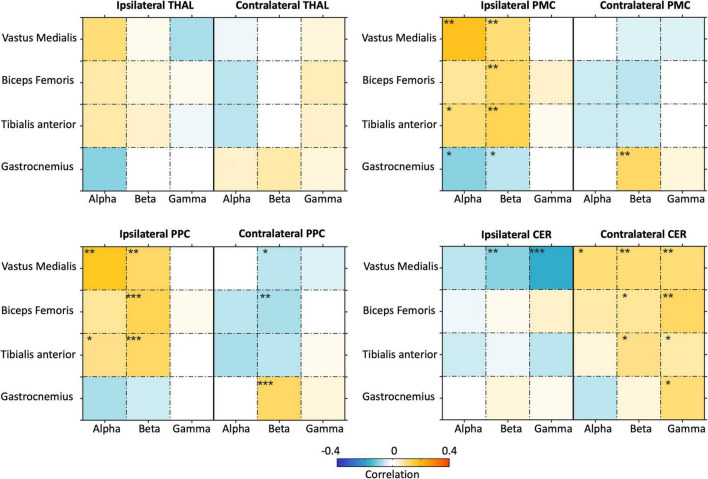
Analysis of brain-body connectivity for ipsilateral and contralateral thalamus (THAL), premotor cortex (PMC), posterior parietal cortex (PPC), and cerebellum (CER), in alpha, beta, and gamma frequency bands. The brain-body connectivity is assessed using envelopes of reconstructed neural signals and EMG envelopes. *p_*FDR*_ < 0.05;**p_FDR_ < 0.01; ***p_FDR_ < 0.001.

Considering that the two NKC and NMC metrics were dependent to each other, and the muscular signals showed relatively lower inter-dependence, we focused on NMC only from this point on. By extending the ROI analysis to THAL, PMC, PPC, and CER, we specifically investigated whether brain-body connectivity involves not only M1, a brain region supporting motor execution, but also other regions typically associated with motor planning and coordination (Figure 9). Overall, we found robust NMC in M1, PMC, PPC, and CER regions, whereas much weaker effects were observed in THAL. The ANOVA results showed the NMC values were strongly modulated (*p*_*FDR*_ < 0.001) on the body sensor in bilateral M1, bilateral PMC, ipsilateral PPC, and ipsilateral CER, and by the frequency band in the ipsilateral M1 only (Supplementary Table 3).

### Whole-Brain Analysis of Brain-Body Connectivity

**FIGURE 10:**
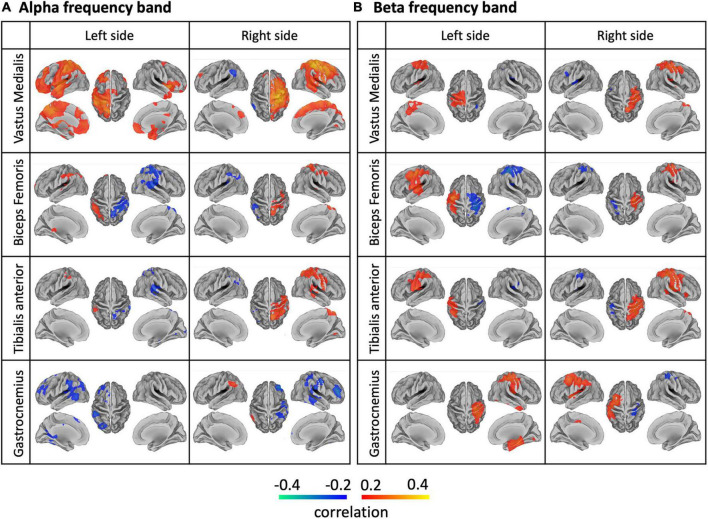
Brain-body connectivity maps for **(A)** alpha and **(B)** beta frequency bands, obtained using EMG envelopes. We considered the EMG envelopes for each of the eight body sensors: left/right vastus medialis, biceps femoris, tibialis anterior, gastrocnemius. We used the threshold *r* > 0.2 for visualization purposes.

A whole-brain analysis was conducted to assess the spatial specificity of the NMC patterns. In general, we observed lateralized NMC patterns for each sensor both in the alpha and beta bands over or close to the primary sensorimotor cortex (Figure 10). For the vastus medialis, the biceps femoris and the tibialis anterior, alpha-band and beta-band NMC had positive values over the ipsilateral hemisphere, and peaked at different locations of the primary sensorimotor cortex; furthermore, the positive alpha-band NMC was more widespread, and therefore less spatially specific, than the beta-band NMC, whereas beta-band NMC was more focal over the ipsilateral sensorimotor cortex. Negative alpha- and beta-band NMC values were primarily found in the contralateral hemisphere, and had less consistent spatial distribution across body sensors than positive NMC values. The alpha- and beta-band NMC for the gastrocnemius appeared to have reversed polarity with respect to that of the other sensors. The NMC maps for the gamma band had lower intensity than for the alpha and beta bands, and were less reliable across body sensors (Supplementary Figure 5).

**FIGURE 11:**
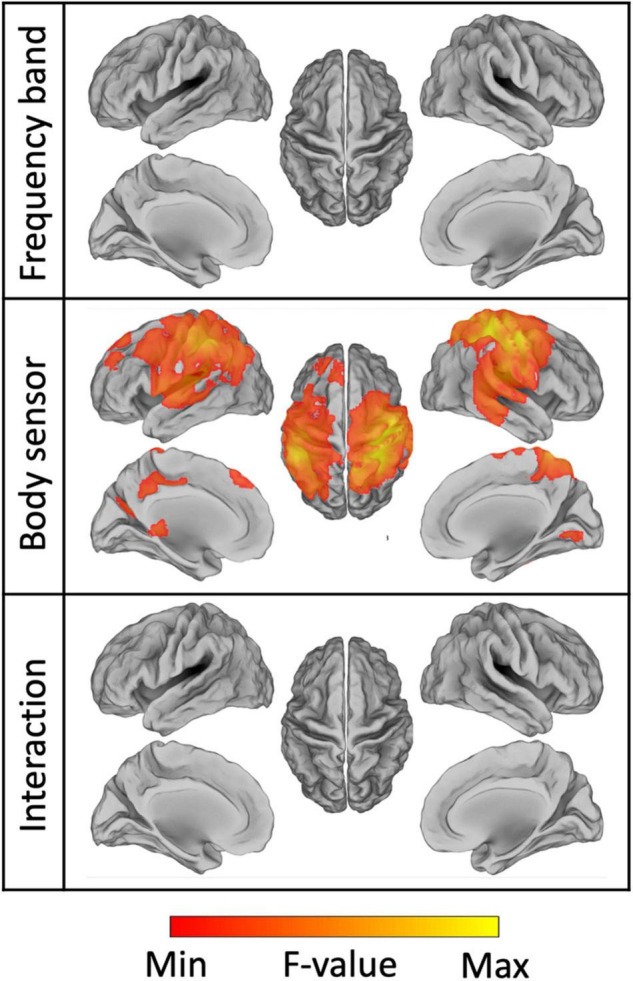
Results of the ANOVA conducted on the source images of brain-body connectivity. We specifically performed a two-way ANOVA, using the frequency band and the body sensors as factors, respectively. The resulting maps for the main effects of frequency band, body sensor, and their interaction are thresholded at *p_FDR_* < 0.001.

To further test the effect of frequency band, body sensor and their interaction, we applied a voxel-wise full factorial ANOVA on the connectivity images (Figure 11). The analysis revealed significant differences (*p_FDR_* < 0.001) in NMC across the body sensors mainly over the bilateral primary sensorimotor cortex, but did not result in any regions with either significant differences across frequency bands (*p_FDR_* > 0.99) or significant interaction between body sensor and frequency band (*p_FDR_* > 0.98). Overall, the ANOVA map obtained for the body sensor as factor spanned not only M1 regions, but included also other regions in the premotor and posterior parietal cortex. These results were largely consistent with what already observed thorough the ROI analyses (Figures 8B, 9 and Supplementary Table 3).

## Discussion

In this study, we investigated brain-body connectivity during gait using a hdEEG-based mobile brain-body imaging platform. Considering that brain-body connectivity can be quantified either using muscular (NMC) or kinematic (NKC) signals, we also evaluated which of the two measures may yield the largest discriminative power. Next, we tested whether gait-related brain-body connectivity is characterized by specific spatial patterns depending on the specific body sensor and the neural oscillations of interest, and we evaluated if the brain regions showing robust gait-related brain-body connectivity are those typically related to motor execution, or also to motor planning and coordination. To the best of our knowledge, this was the first study that addressed these specific questions by examining source-reconstructed EEG signals, rather than EEG recordings. Our results showed that myogenic body signals have more discriminative power than kinematic signals in evaluating gait-related brain-body connectivity, and that brain-body connectivity measures map on brain regions related to motor execution, but also motor planning and coordination. In addition, the gait-related brain-body connectivity showed to be dependent on the body sensor used to extract kinematic/EMG dynamics, and only to a much lesser extent to the frequency of the neural oscillations measured using EEG. We will more extensively discuss these findings in the following sections.

### Kinematic and Myogenic Signals for Brain-Body Connectivity Analysis

We started our investigations by analyzing the kinematic and myogenic signals, respectively. We generally observed significant correlations across body sensors, not only within but also between signal types. This was expected, as the contractions of skeletal muscles are the main drivers of the lower limb velocities and accelerations (van Leeuwen et al., 2003). Several EMG studies provided a detailed characterization of lower-limb muscular activity profiles during normal gait (Winter and Yack, 1987; Liu et al., 2008; Allen et al., 2013; Bonnefoy-Mazure and Armand, 2015). Specifically, it was observed that the leg muscles are activated in a coordinated sequential pattern across the gait cycle, supporting balance and movement control (Bonnefoy-Mazure and Armand, 2015). The EMG envelopes in our study were largely in line with previous findings. For example, the vastus medialis of a heel-striking lower limb was activated from the initial double support phase to the beginning of the stance. The biceps femoris activated from the terminal part of the swing phase until the double support phase, which occurs just after the heel strike. The activity of the tibialis anterior spanned the swing and the subsequent double support phases. The gastrocnemius mainly activated during stance, in opposition to the above-mentioned muscles. Overall, the leg movements can be modeled as two double-pendulums, and accelerations/velocities of the same bone should share a similar pattern (Kubo et al., 2004; Sartori et al., 2014). It is therefore not surprising that we found relatively higher inter-dependence in kinematic signals than in myogenic signals. We therefore concluded that the myogenic signals had more discriminative power, and were more suited to assess brain-body connectivity.

### Characterization of Brain-Body Connectivity Images

We characterized gait-related brain-body connectivity in the whole brain by generating source images of NMC for each frequency band across body sensors. Reliable connectivity images were generally obtained with neural oscillations in the alpha and beta bands. This is consistent with several studies that reported modulations of alpha and beta oscillations during movement performance, both in seated conditions (Pfurtscheller et al., 1996; Tan et al., 2016; Porcaro et al., 2018, 2021; Zhao et al., 2019) and walking conditions (Wagner et al., 2016; Zhao et al., 2022). Strongest brain-body connectivity values were located over or close to the bilateral M1, the portion of cortex that is primarily associated with movement execution (Todorov, 2000; Lemon, 2008). Brain regions that are typically associated with motor planning and coordination, as for instance PMC, PPC, and CER (Battaglia-Mayer and Caminiti, 2019; Zhao et al., 2022), showed less strong but still significant NMC values. Furthermore, the voxel-wise ANOVA that we conducted in our study revealed significant differences in brain-body connectivity across body sensors over the bilateral primary sensorimotor cortex. This indicates that the leg muscles connect with the brain in a somatotopic manner (Artoni et al., 2017), and that the activity of the brain regions involved may be temporally modulated to support sophisticated muscular actions of gait performance (Neptune et al., 2004; Liu et al., 2008; Bonnefoy-Mazure and Armand, 2015). The ANOVA on NMC maps revealed no significant difference across frequency bands, and no interaction between frequency bands and sensors. Accordingly, alpha and beta neural oscillations, and to some extent also gamma neuronal oscillations, support the coordinated muscular actions of gait performance (Zhao et al., 2022), but no clear functional dissociation between them could be detected in terms of brain-body connectivity at the whole-brain level.

### Gait-Related Brain-Body Connectivity in Motor-Related Regions

In this study, we also conducted analyses on frequency-dependent brain-body connectivity for several motor-related ROIs, among which M1, THAL, PMC, PPC, and CER. These analyses provided largely coherent results as compared to the whole-bran analyses, showing that myogenic activity is not only related to neural activity in M1, but also PMC, PPC, and CER. The results that we obtained for THAL were much less clear than the other regions. For the bilateral M1, NMC, and NKC values were rather weak for the gamma band, probably because that gamma oscillations are more associated with motor planning (Brovelli et al., 2005; Thürer et al., 2016) and coordination (Santarnecchi et al., 2017; Li et al., 2020) than motor execution. The vastus medialis, biceps femoris, and tibialis anterior showed negative alpha- and beta-band NMC for the contralateral M1. Notably, this finding is in line with one of our previous hdEEG studies, in which the contraction of tibialis anterior yielded a power decrease of alpha- and beta-band neural oscillations in the contralateral hemisphere of the primary sensorimotor cortex (Zhao et al., 2019), which is indicative of an increased excitability of local neurons supporting motor performance (Pfurtscheller and Lopes da Silva, 1999; Neuper et al., 2006). It should be noted that, unlike the other muscles, the gastrocnemius activation during gait correlated with alpha- and beta-band neural power increases in the contralateral M1. Furthermore, leg velocity correlated with decreases in alpha- and beta-band neural power for the contralateral M1. Taking these observations together, we can further infer that the gait-related desynchronization of alpha and beta oscillations may primarily relate to the leg movements, rather than the leg muscle contractions. This may be the case that the fluctuations in the level of recruitment of local neurons in contralateral M1 during walking mainly reflect the changes in uncertainty estimations between sensory predictions and actual sensory inputs of the lower limb movements, rather than being exclusively related to muscle control (Tan et al., 2016).

### Limitations

We should acknowledge that our study has a number of limitations. First, we only included a treadmill walking task. Future investigation of brain-body connectivity in overground free walking conditions are warranted, as they may reveal further information regarding the neural processes of gait control. Second, our analysis of NKC only included velocity signals acquired from the lower limbs. The use a three-dimensional infrared camera system (Pfister et al., 2014) and force measuring treadmill (De Witt and Ploutz-Snyder, 2014) may provide additional data, for instance joint angles and ground reaction forces, that would enable more extensive investigations on NKC; likewise, collecting EMG signals from additional lower limb muscles, as compared to those we included in this study, may be beneficial for future investigations on NMC (Bonnefoy-Mazure and Armand, 2015). Third, the synchronization between EMG and EEG in the MoBI platform was performed offline. It should be considered, however, that real-time synchronization may be desirable for applications out of the research field (King and Parada, 2021). Lastly, we evaluated the NMC and NKC using a trial-averaged approach to increase the signal to noise ratio. The use of trial-level modeling methods (Kamiński et al., 2001; Chen et al., 2021) is warranted in future studies to examine temporal delays between brain and body signals (Artoni et al., 2017; Xu et al., 2017).

## Conclusion

Overall, our study contributed to a finer characterization of brain-body connectivity during gait in healthy individuals, revealing robust relationships between muscular, kinematic, and neural signals. Notably, we performed a characterization of muscular signals considering one EMG sensor at the time. An interesting avenue for future research is extraction of gait-related muscle synergies from multi-channel EMG data (Chia Bejarano et al., 2017), and the analysis of their relationship with neural signals. Future studies may also be directed toward the investigation of different walking conditions, such as passive walking with exoskeleton support (Alqahtani et al., 2021) or gravity-reduced walking (Richter et al., 2021). Furthermore, we believe it would be interesting to use the MoBI approach to simultaneously collect kinematic, EMG and EEG data in individuals with neuromotor disorders such as Parkinson’s disease, to identify neural correlates of abnormal gait (Cao et al., 2021; Tosserams et al., 2022).

## Data Availability Statement

The original contributions presented in the study are included in the article/Supplementary Material, further inquiries can be directed to the corresponding author.

## Ethics Statement

The studies involving human participants were reviewed and approved by Ethics Committee of the Liguria Region, Italy (reference: 238/2019). The patients/participants provided their written informed consent to participate in this study.

## Author Contributions

MZ: experimental design, methodology, data acquisition, data analysis, visualization, and writing. GB: data acquisition and writing. JS and GT: methodology and writing. CP: conceptualization and writing. EP and LA: conceptualization, data acquisition, and writing. DM: conceptualization, experimental design, methodology, visualization, and writing. All authors contributed to the article and approved the submitted version.

## Conflict of Interest

The authors declare that the research was conducted in the absence of any commercial or financial relationships that could be construed as a potential conflict of interest.

## Publisher’s Note

All claims expressed in this article are solely those of the authors and do not necessarily represent those of their affiliated organizations, or those of the publisher, the editors and the reviewers. Any product that may be evaluated in this article, or claim that may be made by its manufacturer, is not guaranteed or endorsed by the publisher.
