# Supplementary material for: Assessing Neurokinematic and Neuromuscular Connectivity During Walking Using Mobile Brain-Body Imaging
Source: Front Neurosci. 2022 Jun 3;16:912075. doi: 10.3389/fnins.2022.912075 (PMC9204106; doi:10.3389/fnins.2022.912075)
Supplement: Supplementary file 1 [file Data_Sheet_1.docx]

Supplementary Material

# Supplementary Figures and Tables

## Supplementary Figures


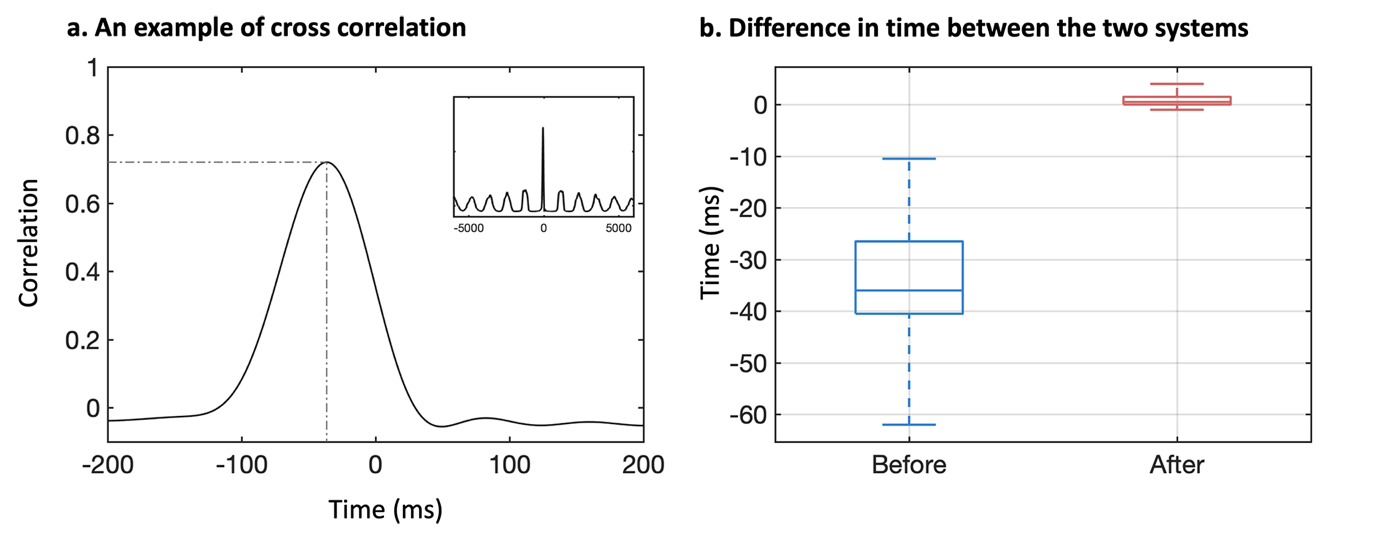


**Figure S1**. Results of the offline synchronization between the timing of the EEG and body sensor devices. (a) An example of cross correlation between signals, used for time-lag detection. (b) Timing difference between the two devices, before and after offline synchronization.


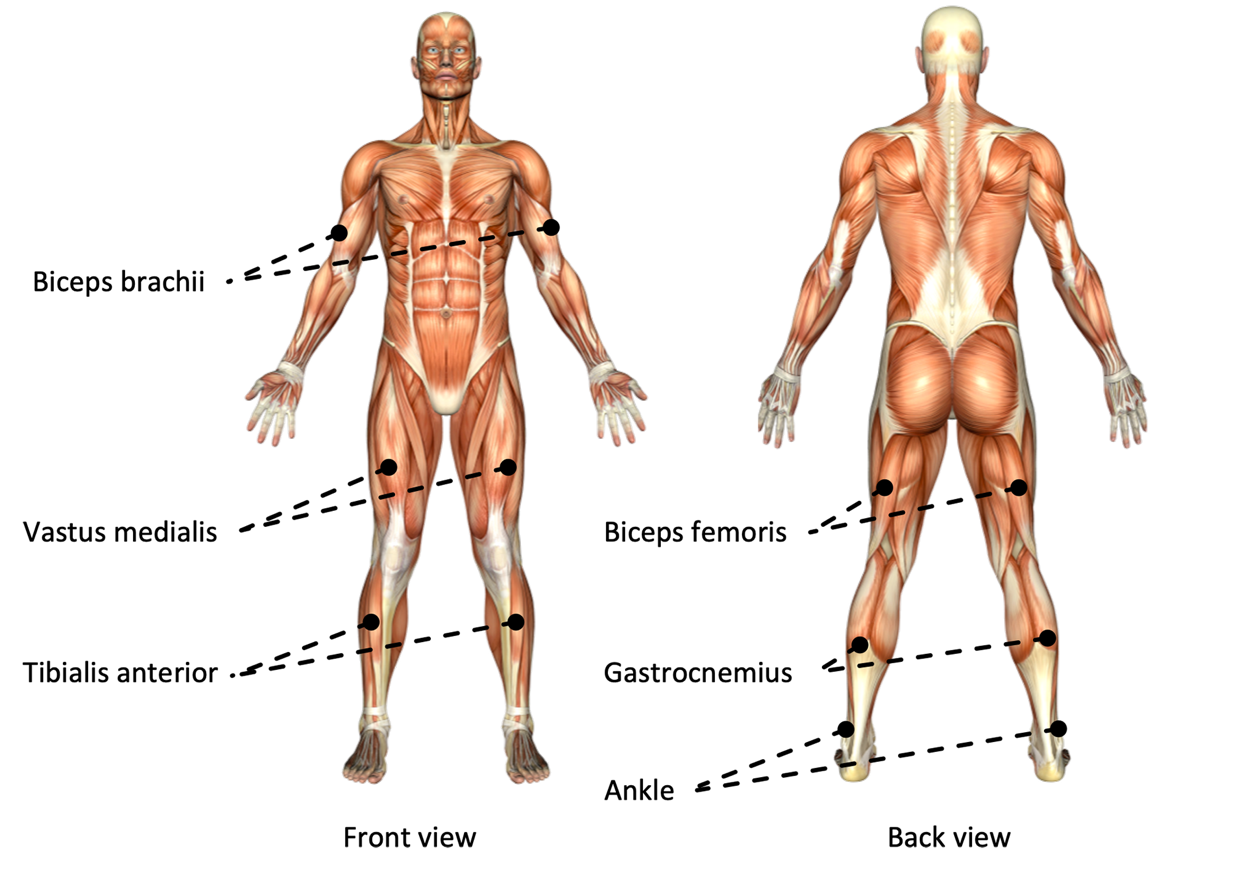


**Figure S2**. Positioning of the wireless body sensors. The muscles over which a body sensor was placed for kinematic and EMG recordings are indicated in the figure.


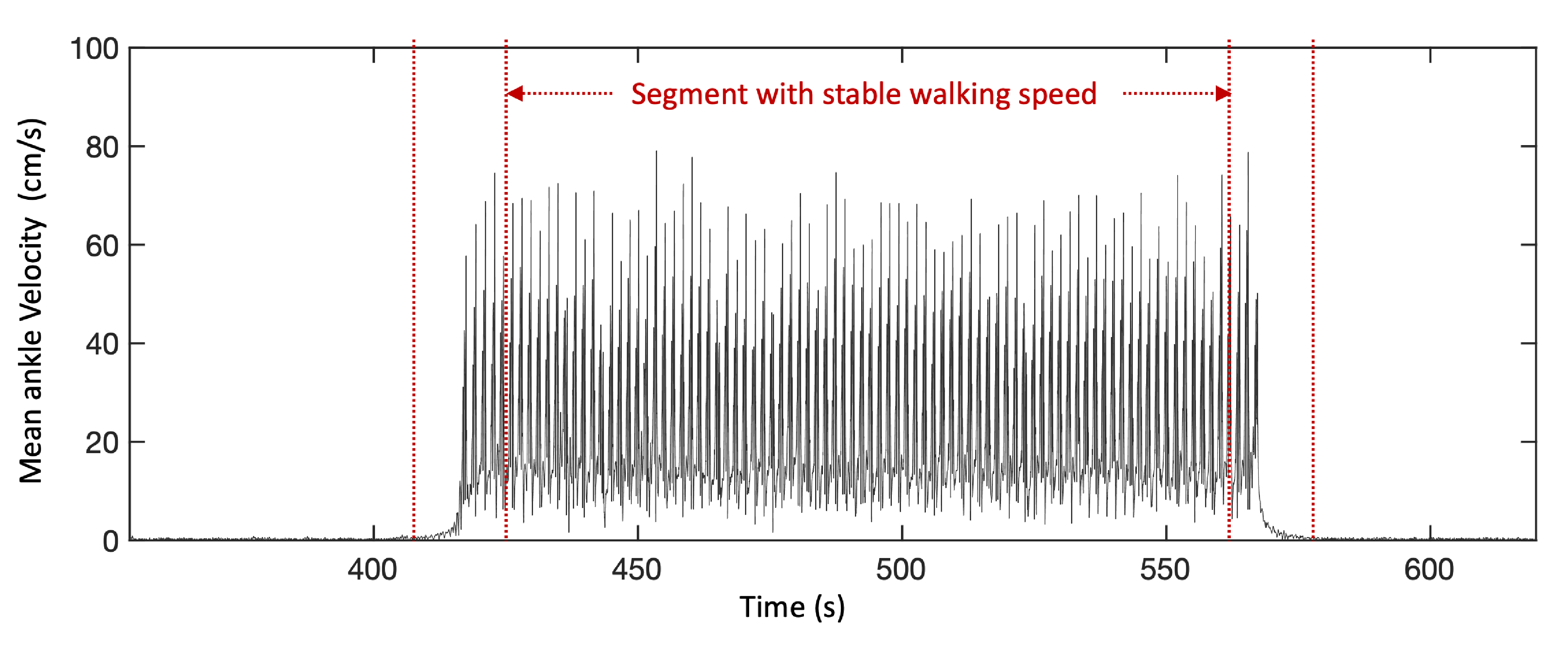


**Figure S3**. Example of ankle velocity trace, with indication of the segment with stable walking speed. The definition of the segment was performed by visual inspection. Only the data segments with stable walking speed were used for behavioral analyses, as well as for brain-body connectivity analyses.


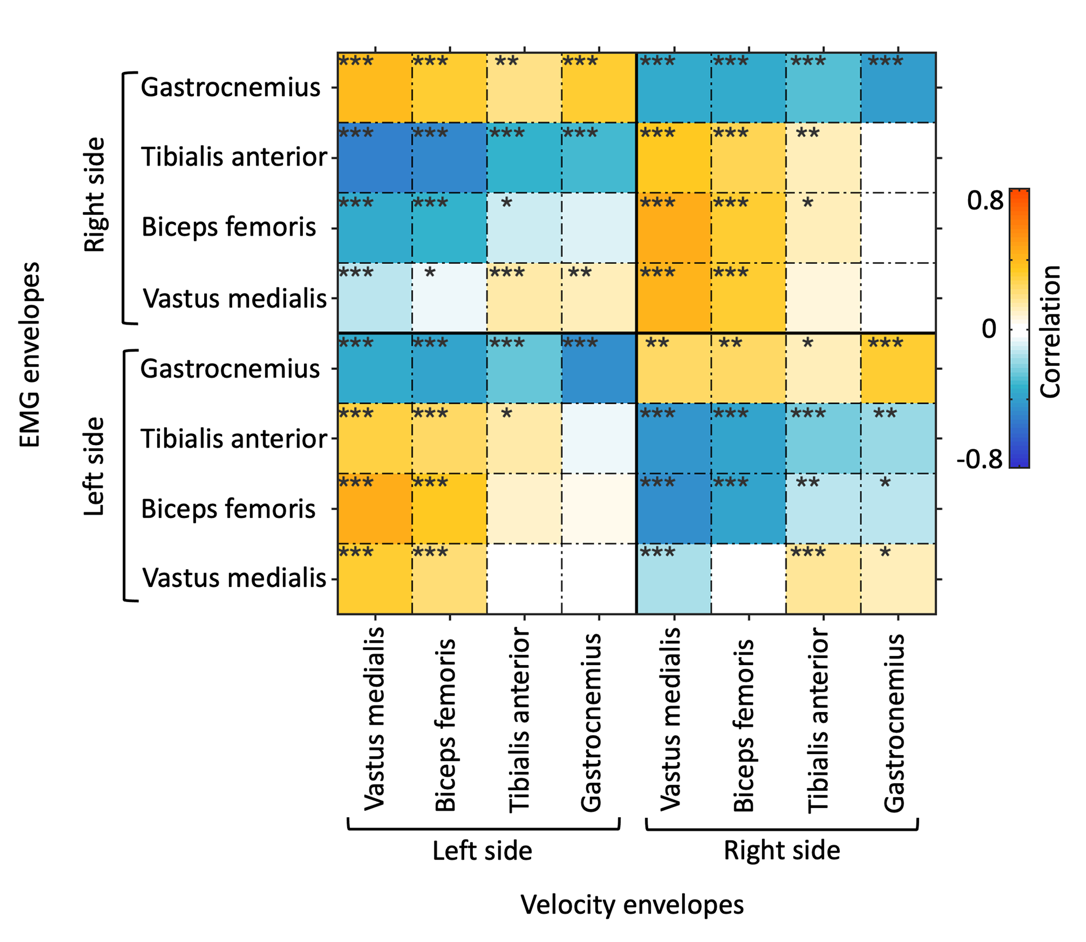


**Figure S4**. Cross-correlations between velocity and EMG envelopes. The results presented refer to the average across participants. *** $p_{FDR}<0.05;\text{**}\text{ }p_{FDR}<0.01\text{;}$ ***** $p_{FDR}<0.001.$


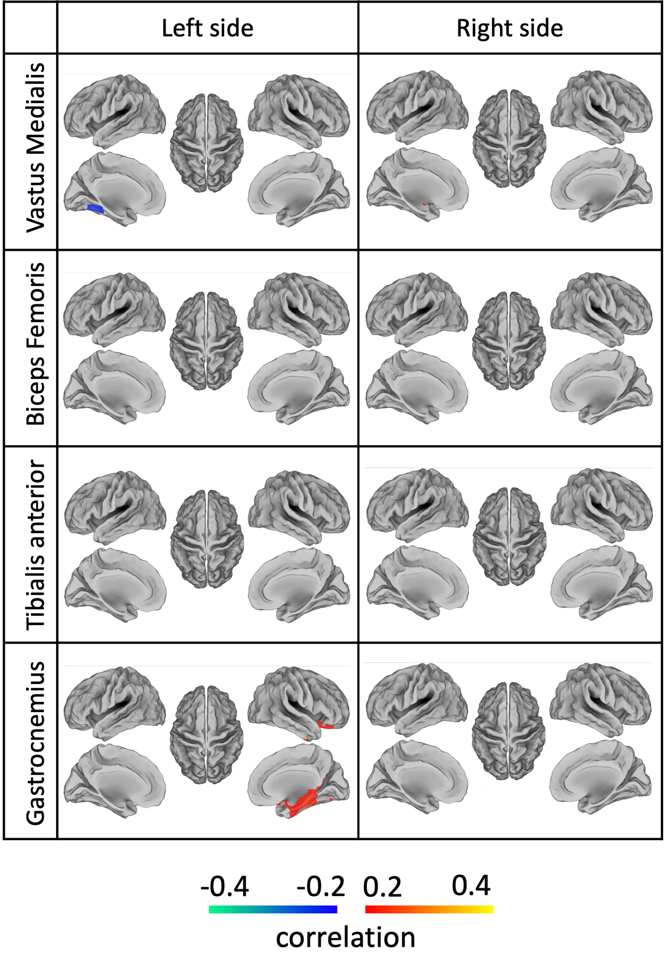


**Figure S5**. Brain-body connectivity maps for the gamma frequency band, obtained using EMG envelopes. We considered the EMG envelopes for each of eight body sensors: left/right vastus medialis, biceps femoris, tibialis anterior, gastrocnemius.

## Supplementary tables

**Table S1**. Positions of the regions of interests (ROI) considered in the study. For each ROI, the region name, the acronym used, and the coordinates in MNI space are provided.

| **Region of interest** | **Acronym** | **MNI coordinates** |
| --- | --- | --- |
| Left/right primary motor cortex | M1 | $[-/+34, -12, 68]$ |
| Left/right thalamus | THAL | $[-/+16 -34, 8]$ |
| Left/right premotor cortex | PMC | $[-/+45, 1, 49]$ |
| Left/right posterior parietal cortex | PPC | $[-/+40, -50, 60]$ |
| Left/right cerebellum | CER | $[-/+42, -73, -49]$ |

**Table S2.** Results of a four-way analysis of variance (ANOVA) on brain body connectivity values. The factors used for this analysis are the following: body sensor (left/right vastus medialis, biceps femoris, tibialis anterior, gastrocnemius), region of interest (ipsilateral M1, contralateral M1), the frequency band (alpha, beta, gamma) and the signal type (EMG envelope, velocity envelope) as factors.

| **Factor** | **S.S.** | **d.f.** | **F** | **p** |
| --- | --- | --- | --- | --- |
| Body sensor | 0.752 | 3 | 6.692 | <0.001 |
| ROI | 7.069 | 1 | 188.733 | <0.001 |
| Frequency band | 1.010 | 2 | 12.488 | <0.001 |
| Signal type | 0.002 | 1 | 0.061 | 0.806 |

**Table S3**. Results of a two-way analysis of variance (ANOVA) on NMC values extracted for different ROIs. The two factors are the body sensor (left/right vastus medialis, biceps femoris, tibialis anterior, gastrocnemius) and the frequency band (alpha, beta, gamma), respectively. The ROIs include ipsilateral and contralateral primary motor cortex (M1), thalamus (THAL), premotor cortex (PMC), posterior parietal cortex (PPC) and cerebellum (CER). p_FDR_: p values corrected for multiple comparison using the false discovery rate (FDR) method.

| **ROI** | **Factor** | **S.S.** | **d.f.** | **F** | **p** | **p_FDR_** |
| --- | --- | --- | --- | --- | --- | --- |
| Ipsilateral M1 | Body sensor | 3.680 | 3 | 26.924 | <0.001 | <0.001 |
|  | Frequency band | 0.960 | 2 | 10.533 | <0.001 | <0.001 |
| Contralateral M1 | Body sensor | 2.258 | 3 | 20.011 | <0.001 | <0.001 |
|  | Frequency band | 0.184 | 2 | 2.447 | 0.087 | 0.111 |
| Ipsilateral THAL | Body sensor | 0.799 | 3 | 4.442 | 0.004 | 0.005 |
|  | Frequency band | 0.416 | 2 | 3.467 | 0.032 | 0.053 |
| Contralateral THAL | Body sensor | 0.502 | 3 | 2.561 | 0.054 | 0.054 |
|  | Frequency band | 0.747 | 2 | 5.718 | 0.004 | 0.012 |
| Ipsilateral PMC | Body sensor | 3.364 | 3 | 16.6158 | <0.001 | <0.001 |
|  | Frequency band | 0.684 | 2 | 5.0692 | 0.007 | 0.016 |
| Contralateral PMC | Body sensor | 1.392 | 3 | 7.247 | <0.001 | <0.001 |
|  | Frequency band | 0.038 | 2 | 0.300 | 0.741 | 0.741 |
| Ipsilateral PPC | Body sensor | 3.062 | 3 | 12.759 | <0.001 | <0.001 |
|  | Frequency band | 0.955 | 2 | 5.968 | 0.003 | 0.012 |
| Contralateral PPC | Body sensor | 0.964 | 3 | 5.248 | 0.001 | 0.002 |
|  | Frequency band | 0.297 | 2 | 2.428 | 0.089 | 0.111 |
| Ipsilateral CER | Body sensor | 2.305 | 3 | 11.754 | <0.001 | <0.001 |
|  | Frequency band | 0.112 | 2 | 0.858 | 0.425 | 0.472 |
| Contralateral CER | Body sensor | 0.735 | 3 | 3.600 | 0.013 | 0.015 |
|  | Frequency band | 0.557 | 2 | 4.093 | 0.017 | 0.034 |
